# Supplementary material for: Establishing an online resource to facilitate global collaboration and inclusion of underrepresented populations: Experience from the MJFF Global Genetic Parkinson’s Disease Project
Source: PLoS One. 2023 Oct 3;18(10):e0292180. doi: 10.1371/journal.pone.0292180 (PMC10547150; doi:10.1371/journal.pone.0292180)

PLOS ONE

Establishing an online resource to facilitate global collaboration and inclusion of underrepresented populations: experience from the MJFF Global Genetic Parkinson's Disease Project

--Manuscript Draft--

Manuscript Number:	PONE-D-22-35560
Article Type:	Research Article
Full Title:	Establishing an online resource to facilitate global collaboration and inclusion of underrepresented populations: experience from the MJFF Global Genetic Parkinson's Disease Project
Short Title:	The MJFF Global Genetic Parkinson's Disease Project Resource
Corresponding Author:	Christine Klein<br>University Hospital Schleswig-Holstein, Campus Luebeck<br>Luebeck, GERMANY
Keywords:	monogenic Parkinson's disease
Abstract:	Parkinson's disease (PD) is the fastest-growing neurodegenerative disorder, currently affecting ~7 million people worldwide. PD is clinically and genetically heterogeneous, with at least 10% of all cases explained by a monogenic cause or strong genetic risk factor. However, the vast majority of our present data on monogenic PD is based on the investigation of patients of European White ancestry, leaving a large knowledge gap on monogenic PD in underrepresented populations. Gene-targeted therapies are being developed at a fast pace and have started entering the clinical trials. In light of these developments, building a global network of centers working on monogenic PD, fostering collaborative research, and establishing a clinical trial-ready cohort is imperative. Based on a systematic review of the English literature on monogenic PD and a successful team science approach, we have built up a network of 59 sites worldwide and have collected information on the availability of data, biomaterials, and facilities. To enable access to this resource and to foster collaboration across centers, as well as between academia and industry, we have developed an interactive map and online tool allowing for a quick overview of available resources, along with an option to filter for specific items of interest. This initiative has recently been merged with the Global Parkinson's Genetics Program (GP2), which will attract additional centers with a focus on underrepresented sites. This growing resource and tool will facilitate collaborative research and impact the development and testing of new therapies for monogenic and potentially for idiopathic PD patients.
Order of Authors:	Eva-Juliane Vollstedt, MD
	Harutyun Madoev
	Anna Aasly
	Azlina Ahmad-Annuar
	Bashayer Al-Mubarak
	Roy N. Alcalay
	Victoria Alvarez
	Ignacio Amorin
	Grazia Annesi
	David Arkadir
	Soraya Bardien
	Roger A. Barker
	Melinda Barkhuizen
	A. Nazli Basak

Powered by Editorial Manager® and ProduXion Manager® from Aries Systems Corporation

	Vincenzo Bonifati
	Agnita Boon
	Laura Brighina
	Kathrin Brockmann
	Andrea Carmine Belin
	Jonathan Carr
	Jordi Clarimon
	Mario Cornejo-Olivas
	Leonor Correia Guedes
	Jean-Christophe Corvol
	David Crosiers
	Joana Damásio
	Parimal Das
	Patricia De Carvalho Aguiar
	Anna De Rosa
	Jolanta Dorszewska
	Sibel Ertan
	Rosangela Ferese
	Joaquim Ferreira
	Emilia Gatto
	Gencer Genc
	Nir Giladi
	Pilar Gómez-Garre
	Hasmet Hanagasi
	Nobutaka Hattori
	Faycal Hentati
	Dorota Hoffman-Zacharska
	Sergey N. Illarioshkin
	Joseph Jankovic
	Silvia Jesús
	Valtteri Kaasinen
	Anneke Kievit
	Péter Klivenyi
	Vladimir Kostic
	Dariusz Koziorowski
	Andrea A. Kühn
	Anthony E. Lang
	Shen-Yang Lim
	Chin-Hsien Lin
	Katja Lohmann
	Vladana Markovic

Powered by Editorial Manager® and ProduXion Manager® from Aries Systems Corporation

	Mika Henrik Martikainen
	George Mellick
	Marcelo Merello
	Lukasz Milanowski
	Pablo Mir
	Özgür Öztop-Cakmak
	Márcia Mattos Gonzalves Pimentel
	Teeratorn Pulkes
	Andreas Puschmann
	Ekaterina Rogaeva
	Esther M. Sammler
	Maria Skaalum Petersen
	Matej Skorvanek
	Mariana Spitz
	Oksana Suchowersky
	Ai Huey Tan
	Pichet Termsarasab
	Avner Thaler
	Vitor Tumas
	Enza Maria Valente
	Bart van de Warrenburg
	Caroline H. Williams-Gray
	Ruey-Mei Wu
	Baorong Zhang
	Alexander Zimprich
	Justin Solle
	Shalini Padmanabhan
	Christine Klein
**Additional Information:**	
**Question**	**Response**
**Financial Disclosure**<br><br>Enter a financial disclosure statement that describes the sources of funding for the work included in this submission. Review the submission guidelines for detailed requirements. View published research articles from *PLOS ONE* for specific examples.<br><br>This statement is required for submission and **will appear in the published article** if	CK received a grant for this project by the Michael J Fox Foundation (ID 15015.03, https://www.michaeljfox.org/).

the submission is accepted. Please make sure it is accurate.<br><br>**Unfunded studies**<br>Enter: *The author(s) received no specific funding for this work.*<br><br>**Funded studies**<br>Enter a statement with the following details:<br>• Initials of the authors who received each award<br>• Grant numbers awarded to each author<br>• The full name of each funder<br>• URL of each funder website<br>• Did the sponsors or funders play any role in the study design, data collection and analysis, decision to publish, or preparation of the manuscript?<br>• **NO** - Include this sentence at the end of your statement: *The funders had no role in study design, data collection and analysis, decision to publish, or preparation of the manuscript.*<br>• **YES** - Specify the role(s) played.<br><br>* typeset	
**Competing Interests**<br><br>Use the instructions below to enter a competing interest statement for this submission. On behalf of all authors, disclose any competing interests that could be perceived to bias this work—acknowledging all financial support and any other relevant financial or non-financial competing interests.<br><br>This statement is required for submission and **will appear in the published article** if the submission is accepted. Please make sure it is accurate and that any funding sources listed in your Funding Information later in the submission form are also declared in your Financial Disclosure statement.<br><br>View published research articles from *PLOS ONE* for specific examples.	I have read the journal's policy and the authors of this manuscript have the following competing interests: Roy N. Alcalay received consulting fees from Avrobio, Caraway, Ono Therapeutics, GSK, Merck, Sanofi, Janssen and grants from the Michael J. Fox Foundation, DOD, the Parkinson's Disease Foundation, and the NIH. Melinda Barkhuizen received a PhD scholarship from the National Research Foundation of South Africa (grant numbers 89230 and 98217) and internal funding from the research center where the study was conducted (DST/NWU Preclinical Drug Development Platform, North-West University, South Africa); Ampath Pathology laboratories in South Africa donated services in the form of drawing participant blood for DNA extractions, Several neurologists in South Africa assisted the authors with identifying patients with Parkinson`s disease and referring them to the genotyping study; the North-West University, South Africa provided Ethical oversight and approval of the genotyping project. Vincenzo Bonifati received grants from Stichting Parkinson Fonds (Netherlands) and Alzheimer Nederland, he received honoraria from the International Parkinson and Movement Disorder Society (MDS), for lectures and as Chair of the International Congress Scientific Program Committee (2020-2021), and from Elsevier Ltd. as co-Editor in Chief of the journal Parkinsonism & Related Disorders (2018-current); he is unpaid member of the Stichting Parkinson Fonds (Netherlands). Kathrin Brockmann received grants from the German Federal Ministry of Education and Research (BMBF; PDStrat; FKZ 031L0137B) and from the German Center for Neurodegenerative Diseases (DZNE), consulting fees from Abbvie, Lundbeck, UCB, Zambon, Roche, and honoraria from Abbvie and UCB. Jordi Clarimon is full-time employee at Lundbeck A/S (Denmark). Mario Cornejo-Olivas has subcontracts with Cleveland Clinic and San Marcos Foundation for recruiting participants for the LARGE PD study in Peru. Jean-Christophe Corvol received grants from Sanofi and the Michael J. Fox Foundation, consulting fees from Biogen, UCB, Denali, Idorsia, Prevail

**NO authors have competing interests**<br><br>Enter: *The authors have declared that no competing interests exist.*<br><br>**Authors with competing interests**<br><br>Enter competing interest details beginning with this statement:<br><br>*I have read the journal's policy and the authors of this manuscript have the following competing interests: [insert competing interests here]*<br><br>* typeset	therapeutics, Theranexus, and honoraria from Biogen. Joana Damásio received honoraria from Zambon Pharmaceuticals. Anna De Rosa received grants for ROPAD – the Rostock International Parkinson's Disease Study, sponsored by Centogene and grants from Zambon and AIFA (Italian Agency of Drug), and she is member of the advisory board at BIAL. Joaquim Ferreira received grants from Fundação MSD (Portugal), Novartis, Medtronic, and Abbvie, and lecture fees from Lundbeck, Abbvie, BIAL, Biogen, Sunovion Pharmaceuticals, ONO, Affiris, and Zambon, payment for expert testimony from Novartis, and he participates in advisory boards of Lundbeck, Abbvie, BIAL, Affiris, Sunovion Pharmaceuticals, and Zambon; he is employed by CNS (Campus Néurologico Sénior) and the Medical Faculty of Lisbon. Emilia Gatto received consulting fees from Bago Argentina, honoraria from Bago Argentina,UCB, IPMDS, and Europharm, and participates in advisory boards of Bago Argentina and UCB. Nir Giladi received grants from the Michael J Fox Foundation, The National Parkinson Foundation, The European Union, The Israel Science Foundation Teva, NNE program, Biogen, Ionis, Sieratzki Family Foundation, and The Aufzien Academic Center in Tel-Aviv University; he holds royalties or licenses at Lysosomal Therapeutics (LTI); he received consulting fees from Sionara, NeuroDerm, Pharma2B, Denali, Neuron23, Sanofi-Genzyme, Biogen, and Abbvie; he received honoraria from Abbvie, Sanofi-Genzyme, and the Movement Disorder Society. Nobutaka Hattori received grants from the Japan Society for the Promotion of Science (JSPS; 18H04043, 21H04820, 19K22603), the Japan Agency for Medical Research and Development (AMED; JP20dm0307101, JP20dm0207070, JP20ek0109358, JP19ek0109393, JP19gm0710011, JP19km0405206), Health Labour Sciences Research Grant (20FC1049, H29-FC1-062, H29-FC1-033), and the Japan Science and Technology Agency (JPMJMS2024-5); he participates in advisory boards at Dai-Nippon Sumitomo Pharma, Takeda Pharmaceutical, Kyowa Kirin, TEIJIN PHARMA LIMITED, Novartis Pharma, Ono Pharmaceutical, Biogen Idec Japan, and Kissei Pharmaceutical; he receives consulting fees from Hisamitsu Pharma; he received honoraria from Dai-Nippon Sumitomo Pharma, Takeda Pharmaceutical, Kyowa Kirin, AbbVie GK, Nippon Boehringer Ingelheim, Otsuka Pharmaceutical, Novartis Pharma, Bristol-Myers Squibb, Ono Pharmaceutical, FP Pharmaceutical, Eisai, Kissei Pharmaceutical, Nihon Medi-physics, and Daiichi Sankyo; he received payment for expert testimony from Mitsubishi Tanabe Pharma; he received support for attending meeting from Takeda Pharmaceutical, and Kyowa Kirin; he has 17 patents planned or pending and 5 issued; he is Team Leader at the Neurodegenerative Disorders Collaborative Laboratory, RIKEN Center for Brain Science; he holds equity stock (8%) of PARKINSON Laboratories Co. Ltd., unrelated to the submitted work. Silvia Jesús has received grants from "Consejería de Salud y Familias" PI-0459-2018, "acción B Clínicos-Investigdores" B-0007-2019 and from the "instituto de Salud Carlos III" PI-18/01898; she received honoraria from Abbvie, Bial, Merz, UCB, Italfarmaco, Zambon and Server. Valtteri Kaasinen received grants from Turku University Foundation, Päivikki and Sakari Sohlberg Foundation, and the Finnish Cultural Foundation; he received consulting fees from Nordic Infucare, Abbvie, and<br>Adamant Health; he received honoraria from Nordic Infucare and Abbvie; he received Support for attending meetings and/or travel from Nordic Infucare; he participates on a Data Safety Monitoring Board or Advisory Board at Nordic Infucare and Abbvie; he is board member of the Finnish Neurological Society. Christine Klein received royalties from Oxford University Press; she received consulting fees from Centogene and Retromer Therapeutics; she received payment or honoraria from Desitin Pharma; she received support to attend meetings from the Movement Disorder Society; she participates on a Data Safety Monitoring Board or Advisory Board at the Else Kroener Fresenius Foundation. Peter Klivenyi received honoraria from Abbvie and Medtronic.

Vladimir Kostic received Grant 17590 from the Ministry of Education, Science and Technological Development of Serbia; he received honoraria from Novartis and Boehringer Ingelheim. Shen-Yang Lim received the following grants, (i) Stipend - Global Parkinson's Genetics Program (GP2) Working Group Co-Lead Award, from the Michael J. Fox Foundation, (ii) Michael J. Fox Foundation (Grant 18305: "Identifying leucocyte and urine biomarkers in PD patients with LRRK2 G2385R variant"). George Mellick received grants from the National Health and Medical Research Foundation, Australia (APP1151854, APP1084560). Lukasz Milanowski received grants from the Polish National Agency for Academic Exchange Iwanowska's Fellowship PPN/IWA/2018/1/00006/U/00001/01, the APDA, the Foundation for Polish Science (FNP) and the Haworth Family Professorship in Neurodegenerative Diseases Fund. Pablo mir received grants from the Spanish Ministry of Science and Innovation (RTC2019-007150-1), the Instituto de Salud Carlos III-Fondo Europeo de Desarrollo Regional (ISCIII-FEDER, PI19/01576), and the Consejería de Salud y Bienestar Social de la Junta de Andalucía (PE-0210-2018); he received payment or honoraria for lectures from Abbvie, Abbott, and Zambon; he received Support for attending meetings and travel from Abbvie. Shalini Padmanabhan is employed by the Michael J. Fox Foundation. Andreas Puschmann received grants from Region Skåne, Sweden, Skåne University Hospital, Sweden, The Swedish Parkinson Academy, The Swedish Parkinson Foundation (Parkinsonfonden), MultiPark – a strategic research environment at Lund University, and Bundy Academy, Sweden; he received honoraria from Elsevier (Associate editor for Parkinsonism and Related Disorders), from the International Parkinson and Movement Disorder Society (MDS), and from the International Association of Parkinsonism and Related Disorders; he received support for travels from the International Association of Parkinsonism and Related Disorders; he is board member of the International Association of Parkinsonism and Related Disorders. Mariana Spitz received funding from Centogene for the ROPAD study; she received support for travels from Roche. Justin Solle is employed by the Michel J. Fox Foundation. Oksana Suchowersky received grants from Roche and WaveLifeSciences; she holds royalties or licenses at UpToDate; she received consulting fees from Abbvie and Sunovion; she received honoraria from the World Parkinson Conference; she participates in advisory boards at Alexion; she is board member of the Parkinson Society Alberta. Pichet Termsarasab received book royalties from Elsevier and Springer Nature Switzerland; he received honoraria for manuscript writing from MedLink Neurology and for lectures from Viatris. Bart van de Warrenburg received grants from Radboudumc, ZonMW, Hersenstichting, Gossweiler Foundation, and the Michael J Fox Foundation; he holds royalties or licenses at BSL Springer Nature; he received consulting fees from uniQure; he received honoraria from UKM Medical Center Kuala Lumpur; he participates in Medical advisory board of patient organizations (unpaid); he is unpaid member of Steering committees or executive boards of various research consortia; he received a wearable sensor set by Brugling Fund/Hersenstichting. Caroline H. Williams-Gray collaborates with Astra-Zeneca on the microbiome in Parkinson's disease; she received consultancy fees from Modus Outcomes and Evidera, Inc./GlaxoSmithKline; she received honoraria from Profile Pharma Limited. Eva-Juliane Vollstedt, Harutyun Madoev, Anna Aasly, Azlina Ahmad-Annuar, Bashayer Al-Mubarak, Victoria Alvarez, Ignacio Amorin, Grazia Annesi, David Arkadir, Soraya Bardien, Roger A. Barker, A. Nazli Basak, Agnita Boon, Laura Brighina, Andrea Carmine Belin, Jonathan Carr, Leonor Correia Guedes, David Crosiers, Parimal Das, Patricia de Carvalho Aguiar, Jolanta Dorszewska, Sibel Ertan, Rosangela Ferese, Gençer Genç, Pilar Gómez-Garre, Hasmet Hanagasi, Faycal Hentati, Dorota Hoffman-Zacharska, Sergey N. Illarioshkin, Joseph Jankovic, Anneke Kievit, Dariusz Koziorowski, Andrea A. Kühn, Anthony E. Lang, Chin-Hsien Lin, Katja

Powered by Editorial Manager® and ProduXion Manager® from Aries Systems Corporation

	Lohmann, Vladana Markovic, Mika Henrik Martikainen, Marcelo Merello, Özgür Öztop-Çakmak, Márcia Mattos Gonçalves Pimentel, Teeratorn Pulkes, Ekaterina Rogaeva, Esther M. Sammler, Maria Skaalum Petersen, Matej Skorvanek, Ai Huey Tan, Avner Thaler, Vitor Tumas, Enza Maria Valente, Ruey-Mei Wu, Baorong Zhang, and Alexander Zimprich report no conflict of interest.
**Ethics Statement**<br><br>Enter an ethics statement for this submission. This statement is required if the study involved:<br><br>• Human participants<br>• Human specimens or tissue<br>• Vertebrate animals or cephalopods<br>• Vertebrate embryos or tissues<br>• Field research<br><br>Write "N/A" if the submission does not require an ethics statement.<br><br>General guidance is provided below. Consult the submission guidelines for detailed instructions. **Make sure that all information entered here is included in the Methods section of the manuscript.**	N/A

**Format for specific study types**<br><br>**Human Subject Research (involving human participants and/or tissue)**<br>• Give the name of the institutional review board or ethics committee that approved the study<br>• Include the approval number and/or a statement indicating approval of this research<br>• Indicate the form of consent obtained (written/oral) or the reason that consent was not obtained (e.g. the data were analyzed anonymously)<br><br>**Animal Research (involving vertebrate animals, embryos or tissues)**<br>• Provide the name of the Institutional Animal Care and Use Committee (IACUC) or other relevant ethics board that reviewed the study protocol, and indicate whether they approved this research or granted a formal waiver of ethical approval<br>• Include an approval number if one was obtained<br>• If the study involved *non-human primates*, add *additional details* about animal welfare and steps taken to ameliorate suffering<br>• If anesthesia, euthanasia, or any kind of animal sacrifice is part of the study, include briefly which substances and/or methods were applied<br><br>**Field Research**<br><br>Include the following details if this study involves the collection of plant, animal, or other materials from a natural setting:<br>• Field permit number<br>• Name of the institution or relevant body that granted permission	
**Data Availability**<br><br>Authors are required to make all data underlying the findings described fully available, without restriction, and from the time of publication. PLOS allows rare exceptions to address legal and ethical concerns. See the PLOS Data Policy and FAQ for detailed information.	Yes - all data are fully available without restriction

A Data Availability Statement describing where the data can be found is required at submission. Your answers to this question constitute the Data Availability Statement and **will be published in the article**, if accepted.<br><br>**Important:** Stating 'data available on request from the author' is not sufficient. If your data are only available upon request, select 'No' for the first question and explain your exceptional situation in the text box.<br><br>Do the authors confirm that all data underlying the findings described in their manuscript are fully available without restriction?	
**Describe where the data may be found in full sentences. If you are copying our sample text, replace any instances of XXX with the appropriate details.**<br><br>• If the data are **held or will be held in a public repository**, include URLs, accession numbers or DOIs. If this information will only be available after acceptance, indicate this by ticking the box below. For example: *All XXX files are available from the XXX database (accession number(s) XXX, XXX.).*<br>• If the data are all contained **within the manuscript and/or Supporting Information files**, enter the following: *All relevant data are within the manuscript and its Supporting Information files.*<br>• If neither of these applies but you are able to provide **details of access elsewhere**, with or without limitations, please do so. For example:<br><br>*Data cannot be shared publicly because of [XXX]. Data are available from the XXX Institutional Data Access / Ethics Committee (contact via XXX) for researchers who meet the criteria for access to confidential data.*<br><br>*The data underlying the results presented in the study are available from (include the name of the third party*	The data underlying the results presented in the study are available from https://gp2networkdev.wpengine.com/monogenic-resource-map/.

and contact information or URL).<br>• This text is appropriate if the data are owned by a third party and authors do not have permission to share the data.<br><br>* typeset	
Additional data availability information:	

Manuscript

Click here to access/download;Manuscript;Vollstedt.PLOSOne.2022.MJFF3

BMJ Global Health

Establishing an online resource to facilitate global collaboration and inclusion of underrepresented populations: experience from the MJFF Global Genetic Parkinson's Disease Project

Eva-Juliane Vollstedt[1], Harutyun Madoev[1], Anna Aasly[2], Azlina Ahmad-Annuar[3], Bashayer Al-Mubarak[4], Roy N. Alcalay[5,35], Victoria Alvarez[6], Ignacio Amorin[7], Grazia Annesi[8], David Arkadir[9], Soraya Bardien[10], Roger A. Barker[11], Melinda Barkhuizen[12], A. Nazli Basak[13], Vincenzo Bonifati[14], Agnita Boon[15], Laura Brighina[16], Kathrin Brockmann[17], Andrea Carmine Belin[18], Jonathan Carr[19], Jordi Clarimon[20], Mario Cornejo-Olivas[21], Leonor Correia Guedes[22], Jean-Christophe Corvol[23], David Crosiers[24], Joana Damásio[25], Parimal Das[26], Patricia de Carvalho Aguiar[27], Anna De Rosa[28], Jolanta Dorszewska[29], Sibel Ertan[30], Rosangela Ferese[31], Joaquim Ferreira[32], Emilia Gatto[33], Gençer Genç[34], Nir Giladi[35], Pilar Gómez-Garre[36], Hasmet Hanagasi[37], Nobutaka Hattori[38], Faycal Hentati[39], Dorota Hoffman-Zacharska[40], Sergey N. Illarioshkin[41], Joseph Jankovic[42], Silvia Jesús[36], Valtteri Kaasinen[43], Anneke Kievit[14], Peter Klivenyi[44], Vladimir Kostic[45], Dariusz Koziorowski[46], Andrea A. Kühn[47], Anthony E. Lang[48], Shen-Yang Lim[49], Chin-Hsien Lin[50], Katja Lohmann[1], Vladana Markovic[45], Mika Henrik Martikainen[43], George Mellick[51], Marcelo Merello[52], Lukasz Milanowski[46], Pablo Mir[36], Özgür Öztop-Çakmak[30], Márcia Mattos Gonçalves Pimentel[53], Teeratorn Pulkes[54], Andreas Puschmann[55], Ekaterina Rogaeva[56], Esther M. Sammler[57], Maria Skaalum Petersen[58], Matej Skorvanek[59,60], Mariana Spitz[61], Oksana Suchowersky[62], Ai Huey Tan[49], Pichet Termsarasab[54], Avner Thaler[35], Vitor Tumas[63], Enza Maria Valente[64], Bart van de Warrenburg[65], Caroline H. Williams-Gray[11], Ruey-Mei Wu[50], Baorong Zhang[66], Alexander Zimprich[67], Justin Solle[68], Shalini Padmanabhan[68], Christine Klein[1]

[1]Institute of Neurogenetics, University of Luebeck, Luebeck, Germany
[2] Department of Neuromedicine and Movement Science, Norwegian University of Science and Technology, Trondheim, Norway
[3] Department of Biomedical Science, Faculty of Medicine, University of Malaya, Kuala Lumpur, Malaysia
[4] Center for Genomic Medicine, Research Centre, King Faisal Specialist Hospital and Research Center, Riyadh, Saudi Arabia
[5] Department of Neurology, Columbia University, New York, New York, USA
[6] Laboratório de Genética, Hospital Universitario Central de Asturias, Oviedo, Asturias, Spain, Instituto de Investigación Sanitaria del Principado de Asturias (ISPA), Oviedo, Spain
[7] Universidad de la Republica Uruguay, Montevideo, Uruguay
[8] Institute of Biomedical Research and Innovation, National Research Council, Cosenza, Italy

[9] Department of Neurology, Hadassah Medical Center and the Hebrew University, Jerusalem, Israel
[10] Division of Molecular Biology and Human Genetics, Faculty of Medicine and Health Sciences, Stellenbosch University, Cape Town, South Africa; South African Medical Research Council/Stellenbosch University Genomics of Brain Disorders Research Unit, Stellenbosch University, Cape Town, South Africa
[11] Department of Clinical Neurosciences, University of Cambridge, Cambridge, United Kingdom
[12] DST/NWU Preclinical Drug Development Platform, North-West University, Potchefstroom, North-West, South Africa
[13] Suna and Inan Kiraç Foundation, Neurodegeneration Research Laboratory, KUTTAM, School of Medicine, Koç University, Istanbul, Turkey
[14] Department of Clinical Genetics, Erasmus MC University Medical Center Rotterdam, Rotterdam, The Netherlands
[15]Department of Neurology, Erasmus MC, University Medical Center Rotterdam, Netherlands
[16] Department of Neurology, Milan Center for Neuroscience, University of Milano-Bicocca/San Gerardo Hospital, Monza, Monza Brianza, Italy
[17] Department of Neurodegenerative Diseases, University of Tuebingen, Tuebingen, Baden Wuerttemberg, Germany; Hertie Institute for Clinical Brain Research and German Centre for Neurodegenerative Diseases, Tuebingen, Baden Wuerttemberg, Germany
[18] Department of Neuroscience, Karolinska Institutet, Stockholm, Sweden
[19] Division of Neurology, Department of Medicine, Faculty of Medicine and Health Sciences, Stellenbosch University, Cape Town, South Africa; South African Medical Research Council/Stellenbosch University Genomics of Brain Disorders Research Unit, Stellenbosch University, Cape Town, South Africa
[20]Department of Neurology, Biomedical Research Institute IIB-Sant Pau, Hospital de la Santa Creu i Sant Pau, Barcelona, Spain; Centro de Investigación Biomédica en Red sobre Enfermedades Neurodegenerativas (CIBERNED), Madrid, Spain
[21] Neurogenetics Research Center, Instituto Nacional de Ciencias Neurologicas, Lima, Peru; Center for Global Health, Universidad Peruana Cayetano Heredia, Lima, Peru
[22]Centro de Estudos Egas Moniz, Faculdade de Medicina, Universidade de Lisboa, Portugal; Instituto de Medicina Molecular João Lobo Antunes, Faculty of Medicine, University of Lisbon, Lisbon, Portugal
[23] Sorbonne University, Paris Brain Institute - ICM, Inserm, CNRS, Assistance Publique Hôpitaux de Paris, Pitié-Salpêtrière Hospital, Department of Neurology, Paris, France
[24] Department of Neurology, Antwerp University Hospital, Edegem, Belgium; Translational Neurosciences, Born Bunge Institute, Faculty of Medicine and Health Sciences, University of Antwerp, Wilrijk, Belgium; Center for Molecular Neurology, VIB, Wilrijk, Belgium
[25] Department of Neurology, Hospital de Santo António - Centro Hospitalar Universitário do Porto, Porto, Portugal; UnIGENe, Instituto de Biologia Molecular e Celular (IBMC), Instituto de Investigação e Inovação em Saúde (i3S), Universidade do Porto, Portugal
[26] Centre for Genetic Disorders, Institute of Science, Banaras Hindu University, Varanasi, Uttar Pradesh, India
[27] Hospital Israelita Albert Einstein, São Paulo, Brazil; Department of Neurology and Neurosurgery, Universidade Federal de São Paulo, São Paulo, Brazil
[28] Department of Neurosciences and Reproductive and Odontostomatological Sciences, Federico II University, Naples, Italy
[29] Laboratory of Neurobiology, Department of Neurology, Poznan University of Medical Sciences, Poznan, Poland
[30] Department of Neurology, School of Medicine, Koç University, Istanbul, Turkey
[31] IRCCS Neuromed, Localita' Camerelle, Pozzilli, Isernia, Italy
[32] Laboratory of Clinical Pharmacology and Therapeutics, University of Lisbon, Lisbon, Portugal; Instituto de Medicina Molecular João Lobo Antunes, Faculty of Medicine, University of Lisbon, Lisbon, Portugal
[33] Movement Disorders, Department of Neurology, Instituto de Neurosciencias Buenos Aires, Buenos Aires, Argentina
[34] Department of Neurology, University of Health Sciences, Şişli Hamidiye Etfal Training and Research Hospital, İstanbul, Turkey
[35] Neurological Institute, Tel-Aviv Medical Center, Tel-Aviv, Israel; Sackler School of Medicine, Sagol School of Neuroscience, Tel-Aviv University, Tel-Aviv, Israel
[36] Unidad de Trastornos del Movimiento, Servicio de Neurología y Neurofisiología Clínica, Instituto de Biomedicina de Sevilla, Hospital Universitario Virgen del Rocío/CSIC/Universidad de Sevilla, Seville, Spain; Centro de Investigación Biomédica en Red sobre Enfermedades Neurodegenerativas (CIBERNED), Madrid, Spain
[37] Department of Neurology, Istanbul Faculty of Medicine, Istanbul University, Istanbul, Turkey
[38] Department of Neurology, Juntendo University School of Medicine, Bunkyo, Tokyo, Japan

[39] Mongi Ben Hmida National Institute of Neurology, Tunis, Tunisia
[40] Institute of Genetics and Biotechnology, Faculty of Biology, University of Warsaw, Warsaw, Poland
[41] Department of Neurogenetics, Research Center of Neurology, Moscow, Russia
[42] Parkinson's Disease Center and Movement Disorders Clinic, Department of Neurology, Baylor College of Medicine, Houston, Texas, USA
[43] Neurocenter, Turku University Hospital, Turku, Finland; Clinical Neurosciences, Faculty of Medicine, University of Turku, Turku, Finland; Department of Neurology, Satasairaala Hospital, Pori, Finland
[44] Department of Neurology, University of Szeged, Szeged, Hungary
[45] Department for Neurodegeneration, Clinic for Neurology UCCS, Medical Faculty, University of Belgrade, Belgrade, Serbia
[46] Department of Neurology, Faculty of Health Science, Medical University in Warsaw, Warsaw, Poland
[47] Movement Disorder and Neuromodulation Unit, Charité, Universitätsmedizin Berlin, Department of Neurology, Campus Mitte, Berlin, Germany
[48] Edmond J. Safra Program in Parkinson's Disease, Division of Neurology, Department of Medicine, University of Toronto, Toronto Western Hospital, Toronto, Ontario, Canada
[49] Division of Neurology, Department of Medicine, and the Mah Pooi Soo & Tan Chin Nam Centre for Parkinson's & Related Disorders, Faculty of Medicine, University of Malaya, Kuala Lumpur, Malaysia
[50] Department of Neurology, National Taiwan University Hospital, National Taiwan University College of Medicine, Taipei, Taiwan
[51] Griffith Institute for Drug Discovery (GRIDD), School of Environment and Science, Griffith University, Brisbane, Queensland, Australia
[52] Sección Movimientos Anormales, Departamento de Neurociencias, Fleni, Buenos Aires, Argentina; Argentine National Scientific and Technological Research Council (CONICET), Argentina; Pontificia Universidad Católica Argentina (UCA), Argentina
[53] Department of Genetics, Institute of Biology Roberto Alcantara Gomes, State University of Rio de Janeiro, Rio de Janeiro, Brazil
[54] Division of Neurology, Department of Medicine, Ramathibodi Hospital, Mahidol University, Rajthevi, Bangkok, Thailand
[55] Department of Neurology, Lund University, Sweden; Department of Neurology, Skåne University Hospital, Lund, Sweden
[56] Tanz Centre for Research in Neurodegenerative Diseases, University of Toronto, Toronto, ON, Canada
[57] Medical Research Council Protein Phosphorylation and Ubiquitylation Unit, University of Dundee, Dundee DD1 5EH, UK Molecular and Clinical Medicine, Ninewells Hospital and Medical School, University of Dundee, Dundee DD1 9SY, UK
[58] Centre of Health Science, University of the Faroe Islands, Tórshavn, Faroe Islands; Department of Occupational Medicine and Public Health, The Faroese Hospital System, Faroe Islands
[59] Pavol Jozef Šafárik University in Košice, Košice, Slovakia;
[60] Department of Neurology, University Hospital L. Pasteur, Kosice, Slovakia
[61] Neurology Service, State University of Rio de Janeiro, Rio de Janeiro, Rio de Janeiro, Brazil
[62] Department of Medicine, Medical Genetics and Pediatrics, University of Alberta, Edmonton, Alberta, Canada
[63] Behavioral and Movement Disorders Section, Ribeirão Preto Medical School, University of São Paulo, Brazil
[64] Department of Molecular Medicine, University of Pavia, Pavia, Italy; Neurogenetics Research Centre, IRCCS Mondino Foundation, Pavia
[65] Department of Neurology, Donders Institute for Brain, Cognition, and Behavior, Radboud University Medical Centre, Nijmegen, The Netherlands
[66] Department of Neurology, Second Affiliated Hospital, College of Medicine, Zhejiang University, Hangzhou, Zhejiang, China
[67] Department of Neurology, Medical University, Vienna, Austria
[68] The Michael J. Fox Foundation for Parkinson's Research, New York, USA

Corresponding author:

Christine Klein, MD

Institute of Neurogenetics,

University of Lübeck

Ratzeburger Allee 160

23538 Lübeck, Germany

christine.klein@neuro.uni-luebeck.de

Word count: 1548 words

Abstract word count: 250 words

Acknowledgments

This project is funded by the Michael J. Fox Foundation. Roger Barker and Caroline Williams-Gray are supported by the NIHR Cambridge Biomedical Research Centre (BRC-1215-20014). The views expressed are those of the authors and not necessarily those of the NIHR or the Department of Health and Social Care.

Competing Interests

Roy N. Alcalay received consulting fees from Avrobio, Caraway, Ono Therapeutics, GSK, Merck, Sanofi, Janssen and grants from the Michael J. Fox Foundation, DOD, the Parkinson's Disease Foundation, and the NIH. Melinda Barkhuizen received a PhD scholarship from the National Research Foundation of South Africa (grant numbers 89230 and 98217) and internal funding from the research center where the study was conducted (DST/NWU Preclinical Drug Development Platform, North-West University, South Africa); Ampath Pathology laboratories in South Africa donated services in the form of drawing participant blood for DNA extractions, Several neurologists in South Africa assisted the authors with identifying patients with Parkinson`s disease and referring them to the genotyping study; the North-West University, South Africa provided Ethical oversight and approval of the genotyping project. Vincenzo

Bonifati received grants from Stichting Parkinson Fonds (Netherlands) and Alzheimer Nederland, he received honoraria from the International Parkinson and Movement Disorder Society (MDS), for lectures and as Chair of the International Congress Scientific Program Committee (2020-2021), and from Elsevier Ltd. as co-Editor in Chief of the journal Parkinsonism & Related Disorders (2018-current); he is unpaid member of the Stichting Parkinson Fonds (Netherlands). Kathrin Brockmann received grants from the German Federal Ministry of Education and Research (BMBF; PDStrat; FKZ 031L0137B) and from the German Center for Neurodegenerative Diseases (DZNE), consulting fees from Abbvie, Lundbeck, UCB, Zambon, Roche, and honoraria from Abbvie and UCB. Jordi Clarimon is full-time employee at Lundbeck A/S (Denmark). Mario Cornejo-Olivas has subcontracts with Cleveland Clinic and San Marcos Foundation for recruiting participants for the LARGE PD study in Peru. Jean-Christophe Corvol received grants from Sanofi and the Michael J. Fox Foundation, consulting fees from Biogen, UCB, Denali, Idorsia, Prevail therapeutics, Theranexus, and honoraria from Biogen. Joana Damásio received honoraria from Zambon Pharmaceuticals. Anna De Rosa received grants for ROPAD – the Rostock International Parkinson's Disease Study, sponsored by Centogene and grants from Zambon and AIFA (Italian Agency of Drug), and she is member of the advisory board at BIAL. Joaquim Ferreira received grants from Fundação MSD (Portugal), Novartis, Medtronic, and Abbvie, and lecture fees from Lundbeck, Abbvie, BIAL, Biogen, Sunovion Pharmaceuticals, ONO, Affiris, and Zambon, payment for expert testimony from Novartis, and he participates in advisory boards of Lundbeck, Abbvie, BIAL, Affiris, Sunovion Pharmaceuticals, and Zambon; he is employed by CNS (Campus Néurologico Sénior) and the Medical Faculty of Lisbon. Emilia Gatto received consulting fees from Bago Argentina, honoraria from Bago Argentina,UCB, IPMDS, and Europharm, and participates in advisory boards of Bago Argentina and UCB. Nir Giladi received grants from the Michael J Fox Foundation, The National Parkinson Foundation, The European Union, The Israel Science Foundation Teva, NNE program, Biogen, Ionis, Sieratzki Family Foundation, and The Aufzien Academic Center in Tel-Aviv University; he holds royalties or licenses at Lysosomal Therapeutics (LTI); he received consulting fees from Sionara, NeuroDerm, Pharma2B, Denali, Neuron23, Sanofi-Genzyme, Biogen, and Abbvie; he received honoraria from Abbvie, Sanofi-Genzyme, and the Movement Disorder Society. Nobutaka Hattori received grants from the Japan Society for the Promotion of Science (JSPS; 18H04043, 21H04820, 19K22603), the Japan Agency for Medical Research and Development (AMED; JP20dm0307101, JP20dm0207070,

JP20ek0109358, JP19ek0109393, JP19gm0710011, JP19km0405206), Health Labour Sciences Research Grant (20FC1049, H29-FC1-062, H29-FC1-033), and the Japan Science and Technology Agency (JPMJMS2024-5); he participates in advisory boards at Dai-Nippon Sumitomo Pharma, Takeda Pharmaceutical, Kyowa Kirin, TEIJIN PHARMA LIMITED, Novartis Pharma, Ono Pharmaceutical, Biogen Idec Japan, and Kissei Pharmaceutical; he receives consulting fees from Hisamitsu Pharma; he received honoraria from Dai-Nippon Sumitomo Pharma, Takeda Pharmaceutical, Kyowa Kirin, AbbVie GK, Nippon Boehringer Ingelheim, Otsuka Pharmaceutical, Novartis Pharma, Bristol-Myers Squibb, Ono Pharmaceutical, FP Pharmaceutical, Eisai, Kissei Pharmaceutical, Nihon Medi-physics, and Daiichi Sankyo; he received payment for expert testimony from Mitsubishi Tanabe Pharma; he received support for attending meeting from Takeda Pharmaceutical, and Kyowa Kirin; he has 17 patents planned or pending and 5 issued; he is Team Leader at the Neurodegenerative Disorders Collaborative Laboratory, RIKEN Center for Brain Science; he holds equity stock (8%) of PARKINSON Laboratories Co. Ltd., unrelated to the submitted work. Silvia Jesús has received grants from “Consejería de Salud y Familias” PI-0459-2018, “acción B Clínicos-Investigdores” B-0007-2019 and from the “instituto de Salud Carlos III” PI-18/01898; she received honoraria from Abbvie, Bial, Merz, UCB, Italfarmaco, Zambon and Server. Valtteri Kaasinen received grants from Turku University Foundation, Päivikki and Sakari Sohlberg Foundation, and the Finnish Cultural Foundation; he received consulting fees from Nordic Infucare, Abbvie, and Adamant Health; he received honoraria from Nordic Infucare and Abbvie; he received Support for attending meetings and/or travel from Nordic Infucare; he participates on a Data Safety Monitoring Board or Advisory Board at Nordic Infucare and Abbvie; he is board member of the Finnish Neurological Society. Christine Klein received royalties from Oxford University Press; she received consulting fees from Centogene and Retromer Therapeutics; she received payment or honoraria from Desitin Pharma; she received support to attend meetings from the Movement Disorder Society; she participates on a Data Safety Monitoring Board or Advisory Board at the Else Kroener Fresenius Foundation. Peter Klivenyi received honoraria from Abbvie and Medtronic. Vladimir Kostic received Grant 17590 from the Ministry of Education, Science and Technological Development of Serbia; he received honoraria from Novartis and Boehringer Ingelheim. Shen-Yang Lim received the following grants, (i) Stipend - Global Parkinson's Genetics Program (GP2) Working Group Co-Lead Award, from the Michael J. Fox Foundation, (ii) Michael J. Fox Foundation (Grant 18305: "Identifying leucocyte and urine

biomarkers in PD patients with LRRK2 G2385R variant"). George Mellick received grants from the National Health and Medical Research Foundation, Australia (APP1151854, APP1084560). Lukasz Milanowski received grants from the Polish National Agency for Academic Exchange Iwanowska's Fellowship PPN/IWA/2018/1/00006/U/00001/01, the APDA, the Foundation for Polish Science (FNP) and the Haworth Family Professorship in Neurodegenerative Diseases Fund. Pablo mir received grants from the Spanish Ministry of Science and Innovation (RTC2019-007150-1), the Instituto de Salud Carlos III-Fondo Europeo de Desarrollo Regional (ISCIII-FEDER, PI19/01576), and the Consejería de Salud y Bienestar Social de la Junta de Andalucía (PE-0210-2018); he received payment or honoraria for lectures from Abbvie, Abbott, and Zambon; he received Support for attending meetings and travel from Abbvie. Shalini Padmanabhan is employed by the Michael J. Fox Foundation. Andreas Puschmann received grants from Region Skåne, Sweden, Skåne University Hospital, Sweden, The Swedish Parkinson Academy, The Swedish Parkinson Foundation (Parkinsonfonden), MultiPark – a strategic research environment at Lund University, and Bundy Academy, Sweden; he received honoraria from Elsevier (Associate editor for Parkinsonism and Related Disorders), from the International Parkinson and Movement Disorder Society (MDS), and from the International Association of Parkinsonism and Related Disorders; he received support for travels from the International Association of Parkinsonism and Related Disorders; he is board member of the International Association of Parkinsonism and Related Disorders. Mariana Spitz received funding from Centogene for the ROPAD study; she received support for travels from Roche. Justin Solle is employed by the Michel J. Fox Foundation. Oksana Suchowersky received grants from Roche and WaveLifeSciences; she holds royalties or licenses at UpToDate; she received consulting fees from Abbvie and Sunovion; she received honoraria from the World Parkinson Conference; she participates in advisory boards at Alexion; she is board member of the Parkinson Society Alberta. Pichet Termsarasab received book royalties from Elsevier and Springer Nature Switzerland; he received honoraria for manuscript writing from MedLink Neurology and for lectures from Viatris. Bart van de Warrenburg received grants from Radboudumc, ZonMW, Hersenstichting, Gossweiler Foundation, and the Michael J Fox Foundation; he holds royalties or licenses at BSL Springer Nature; he received consulting fees from uniQure; he received honoraria from UKM Medical Center Kuala Lumpur; he participates in Medical advisory board of patient organizations (unpaid); he is unpaid member of Steering committees or executive boards of various research consortia; he received a wearable sensor

set by Brugling Fund/Hersenstichting. Caroline H. Williams-Gray collaborates with Astra-Zeneca on the microbiome in Parkinson's disease; she received consultancy fees from Modus Outcomes and Evidera, Inc./GlaxoSmithKline; she received honoraria from Profile Pharma Limited. Eva-Juliane Vollstedt, Harutyun Madoev, Anna Aasly, Azlina Ahmad-Annuar, Bashayer Al-Mubarak, Victoria Alvarez, Ignacio Amorin, Grazia Annesi, David Arkadir, Soraya Bardien, Roger A. Barker, A. Nazli Basak, Agnita Boon, Laura Brighina, Andrea Carmine Belin, Jonathan Carr, Leonor Correia Guedes, David Crosiers, Parimal Das, Patricia de Carvalho Aguiar, Jolanta Dorszewska, Sibel Ertan, Rosangela Ferese, Gençer Genç, Pilar Gómez-Garre, Hasmet Hanagasi, Faycal Hentati, Dorota Hoffman-Zacharska, Sergey N. Illarioshkin, Joseph Jankovic, Anneke Kievit, Dariusz Koziorowski, Andrea A. Kühn, Anthony E. Lang, Chin-Hsien Lin, Katja Lohmann, Vladana Markovic, Mika Henrik Martikainen, Marcelo Merello, Özgür Öztop-Çakmak, Márcia Mattos Gonçalves Pimentel, Teeratorn Pulkes, Ekaterina Rogaeva, Esther M. Sammler, Maria Skaalum Petersen, Matej Skorvanek, Ai Huey Tan, Avner Thaler, Vitor Tumas, Enza Maria Valente, Ruey-Mei Wu, Baorong Zhang, and Alexander Zimprich report no conflict of interest.

Abstract

Parkinson’s disease (PD) is the fastest-growing neurodegenerative disorder, currently affecting ~7 million people worldwide. PD is clinically and genetically heterogeneous, with at least 10% of all cases explained by a monogenic cause or strong genetic risk factor. However, the vast majority of our present data on monogenic PD is based on the investigation of patients of European White ancestry, leaving a large knowledge gap on monogenic PD in underrepresented populations. Gene-targeted therapies are being developed at a fast pace and have started entering the clinical trials. In light of these developments, building a global network of centers working on monogenic PD, fostering collaborative research, and establishing a clinical trial-ready cohort is imperative. Based on a systematic review of the English literature on monogenic PD and a successful team science approach, we have built up a network of 59 sites worldwide and have collected information on the availability of data, biomaterials, and facilities. To enable access to this resource and to foster collaboration across centers, as well as between academia and industry, we have developed an interactive map and online tool allowing for a quick overview of available resources, along with an option to filter for specific items of interest. This initiative has recently been merged with the Global Parkinson’s Genetics Program (GP2), which will attract additional centers with a focus on underrepresented sites. This growing resource and tool will facilitate collaborative research and impact the development and testing of new therapies for monogenic and potentially for idiopathic PD patients.

Introduction

As technologies for genetic testing are rapidly developing and are becoming more cost-effective, rare disorders and also rare subtypes of common disorders can increasingly be characterized genetically and thus stratified into genetically defined subgroups. This provides the basis not only to investigate disease mechanisms further but also to develop and test gene-targeted therapies. Parkinson’s disease (PD), the most rapidly growing neurodegenerative disorder (1) can serve as a model disease for this approach, as PD is a common disorder affecting about 7 million people worldwide in 2022, including an estimated 700,000-840,000 (10-12%) hereditary cases with pathogenic genetic variants (monogenic PD and PD due to strong genetic risk factors). In light of the development of gene-targeted therapies for PD, identifying and characterizing carriers of specific genetic pathogenic variants is imperative.

Only a small fraction of the estimated number of subjects with monogenic PD worldwide are currently represented in the literature (2–5). Most study centers publishing in the English language are located in Europe and North America (6). There is evidence for variable penetrance and expressivity of the disease across populations and ethnicities in monogenic forms of PD (2). However, the majority of published cases are reported to be White and originate from Europe or North America, whereas most African, Asian, and South American populations, especially indigenous people, are highly underrepresented. Little is known about the actual worldwide epidemiology, frequency of population-specific variants, and possible local factors influencing the manifestation and course of the disease. Notably, even for published cases, the availability of detailed genetic and clinical data is limited, and genetically stratified clinical trial-ready cohorts are highly sought after.

Global collaboration both within academic research and with the pharmaceutical industry is paramount to expanding our knowledge of disease mechanisms, developing gene-targeted medications, and testing them in clinical trials. Building an international network of researchers from academia and industry will facilitate including more underrepresented populations in investigations and trials, both by enhancing the visibility of existing study centers and by providing genetic testing facilities. To open this network to new members and specifically allow researchers or clinicians from currently underrepresented countries to

engage in these collaborative efforts, we established an online resource that provides an overview of centers worldwide working on monogenic PD and their facilities. While this effort sprung from the MJFF Global Genetic Parkinson's Disease Project (6), it has recently been integrated into the Global Parkinson's Genetics Program (GP2, https://gp2.org/), which will build global will and capacity while addressing emerging research questions in PD genetics (7).

Methods

How to find eligible centers?

Performing a systematic literature review on monogenic PD in the English literature (3,4), our group identified the need for a new, systematic, and global approach to obtain more detailed clinical and genetic data from individuals with monogenic PD. Starting in March 2018, we contacted by email all corresponding authors from articles that we had included in the review and ran an online survey to collect information on the number of subjects with monogenic PD they follow, along with available clinical and genetic data. In a second step, we invited all researchers to provide individual case-level data and offered a small per-case fee to reimburse them for their effort to retrieve and enter the data in another online survey. The vast majority of participating centers expressed enthusiasm about our project, their strong interest in being part of the emerging study group, and support for further collaborative efforts.

To establish the online resource, we contacted all participating researchers, including their collaborators (n=112). We received responses from 59 centers (53%) in 36 countries on all inhabited continents (Europe (n=33), North (n=4) and South America (n=8), Asia (n=10), Australia (n=1), Africa (n=3)). All centers have agreed to be contacted by researchers and the pharmaceutical industry, while the majority of centers (n=41, 69%) would prefer not to be contacted directly but to have requests filtered first. The Monogenic Network of GP2 will handle collaboration requests.

How to obtain the data of interest?

An essential part of this project was communication and information. Having identified eligible researchers, we sent out information emails about our project and kept all participants updated in regular progress emails. To further improve the retention rate, we ensured a quick response in case of any queries and consistently named a specific contact person. When designing the survey, we aimed at providing an accessible online format, allowing different

browser types and devices (again, our team was available and provided help in case of any technical difficulties), kept it brief, and phrased questions in a concise and explicit way (Table 1). Whenever possible, questions were designed to allow single-choice answers only, and free text fields were limited to maximize comparison between centers. For convenience, already available information (such as the name and location of the study center) was prefilled by our team. Participants received an invitation email including a link to their individual survey. Once complete, the survey was submitted by the participant. We sent out two rounds of reminder emails, personally addressing each participant, to improve the response rate and make sure all invitees received their email and got the opportunity to present their study center.

After completing the survey, we reviewed the data and double-checked for any inaccurate entries. In case of ambiguity, we reached out to the respective researcher for approval of the correction. After this first round of quality checks, we the online tool and uploaded the provided data. In a second round of quality checks and to allow for possible updates, we invited all participants to review and verify the provided data and obtained permission to publish the presented resource. Regular updates of the resource and map will be provided biannually, and ongoing projects and trials using the network will be listed on the website to facilitate further collaboration while avoiding duplication of efforts.

Results

Our two-part online tool is now available at https://gp2networkdev.wpengine.com/monogenic-resource-map/. Firstly, it includes a searchable online table allows for filtering for specific characteristics of interest (e.g., location of the center, available facilities, Table 1). Second, a map displays the locations of all research centers and provides individual profiles with information on each center (Figures 1A-C).

Figure 1: Figure 1A is a view of the world map displaying the distribution of all participating centers worldwide. The red pins mark the location of the study center (Figure 1A). Clicking on a red pin opens the respective study center profile (Figures 2B and C). Each profile summarizes information on the site and availability of demographic and clinical data, biomaterials,

methods, and facilities and provides information on other collaborative projects that the study center is part of.

The average number of new PD patients per year reported by the participants until May 2022 is 196. Genetic testing is regularly performed by 37 (63%) of the centers, and the costs are predominantly covered by in-house or external funding sources (39%), followed by coverage by health insurance and the patients themselves (10% each). The availability of biomaterials varies depending on how difficult they are to obtain, ranging from 53 centers (90%) reporting the availability of DNA for at least a subset of cases to 8 centers (14%) reporting the availability of brain tissue (Figure 2). Laboratory facilities that are part of the standard clinical workup like serum and plasma handling, are more frequently available (at 53 centers, 90%) than more specific ones that are more costly or require special training, such as the generation of induced pluripotent stem cells (at 24 centers, 41%; Figure 3).

Figure 2: Availability of biomaterials

Number of centers reporting availability of the listed biomaterials.

DNA: Deoxyribonucleic acid, RNA: Ribonucleic acid, CSF: cerebrospinal fluid, iPSCs: induced pluripotent stem cells

Figure 3: Availability of methods and facilities

Number of centers reporting availability of the listed methods and facilities.

CSF: cerebrospinal fluid, iPSCs: induced pluripotent stem cells, MRI: magnetic resonance imaging, SPECT/PET: Single-photon emission computed tomography/positron emission tomography, TCS: transcranial sonography

Discussion and conclusions

Using a powerful team science approach, we have established a global network of academic centers involved in the clinical care of patients with monogenic PD and with a high interest in collaboration, research, and gene-targeted therapies for PD. Based on a systematic search of the world literature on monogenic PD published in the English language, we have tried to be as inclusive as possible. We have set up the project to serve as a growing resource open to all interested participants. Having focused our initial approach on articles in English is a limitation, as we likely missed centers engaged in the care of patients with monogenic PD who did not

publish on this topic in English. In order to actively broaden this network and to reach out to active centers that have not yet published on monogenic PD, the MJFF Global Genetic Parkinson's Disease Project is currently being merged within GP2, which presently includes 119 centers that will be approached to join this effort if not yet part of the network. Information on GP2 is already available in several languages (https://gp2networkdev.wpengine.com/monogenic-resource-map/), including Arabic, Mandarin, French, German, Japanese, and Spanish. Through this merger, we expect the resource to proliferate and, most importantly, to include an increasing number of previously underrepresented populations and centers.

To facilitate access to this resource and to foster collaboration across centers, as well as between academia and industry, we have developed an interactive map and online tool allowing for a quick overview and descriptive statistics on available data, materials, and facilities, as well as for filtering for specific items of interest, for example, centers who have induced pluripotent stem cell (iPSC) lines of *PRKN* mutation carriers.

We will exploit and expand existing networks through this project and resource to make more clinical and research centers visible and study subjects available to the clinical and basic PD research community. Furthermore, such studies will spur collaborations across global clinical sites and between academia and industry, thus facilitating and expediting the testing of new therapies in relevant PD genetic subtypes. Given the emerging knowledge about disease mechanisms contributing to PD pathogenesis, our hope is that different subpopulations of idiopathic PD subjects would also benefit from these large genetic cohort-based efforts.

Table 1: Items assessed in the online survey

PD: Parkinson's disease; UPDRS: Unified Parkinson's Disease Rating Scale; IRB: institutional review board; DNA: Deoxyribonucleic acid; RNA: Ribonucleic acid; CSF: cerebrospinal fluid; iPSCs: induced pluripotent stem cells; MRI: magnetic resonance imaging, SPECT/PET: Single-photon emission computed tomography/positron emission tomography, TCS: transcranial sonography

**Site details**	Name of site
	Principal investigator
	City/ Country
	Active in other PD projects
	New PD patients/year
	Genetic testing available
	How are genetic testing costs covered?
	Qualified genetic counseling available?
	Already performing clinical trials?
**Availability of clinical data**	Sex
	Age
	Age at onset
	UPDRS
	Hoehn and Yahr scale
	Nonmotor scales
	Olfactory testing
**Data sharing**	IRB consent
	Shipment outside the country
**Availability of biomaterials**	DNA/RNA/Serum/Plasma/whole blood/CSF/fibroblasts/iPSCs/brain tissue (storage?)
**Availability of Omics**	Genomics/ Transcriptomics/ Proteomics/ Metabolomics
**Laboratory facilities**	CSF handling
	Serum Plasma handling
	Culturing of fibroblasts
	Isolation of cells from whole blood
	Generation of iPSCs
	Neuropathology
	MRI
	SPECT/PET
	TCS

References

1. Bloem BR, Okun MS, Klein C. Parkinson’s disease. The Lancet [Internet]. 2021 Apr; Available from: https://linkinghub.elsevier.com/retrieve/pii/S014067362100218X
2. Hentati F, Trinh J, Thompson C, Nosova E, Farrer MJ, Aasly JO. LRRK2 parkinsonism in Tunisia and Norway: A comparative analysis of disease penetrance. Neurology [Internet]. 2014 Aug 5;83(6):568–9. Available from: http://www.neurology.org/cgi/doi/10.1212/WNL.0000000000000675
3. Kasten M, Hartmann C, Hampf J, Schaake S, Westenberger A, Vollstedt EJ, et al. Genotype-Phenotype Relations for the Parkinson’s Disease Genes Parkin, PINK1, DJ1: MDSGene Systematic Review. Movement Disorders [Internet]. 2018 May 11;33(5):730–41. Available from: http://doi.wiley.com/10.1002/mds.27352
4. Trinh J, Zeldenrust FMJ, Huang J, Kasten M, Schaake S, Petkovic S, et al. Genotype-phenotype relations for the Parkinson’s disease genes SNCA, LRRK2, VPS35: MDSGene systematic review. Mov Disord [Internet]. 2018 Oct 24; Available from: http://www.ncbi.nlm.nih.gov/pubmed/30357936
5. Tolosa E, Vila M, Klein C, Rascol O. LRRK2 in Parkinson disease: challenges of clinical trials. Nat Rev Neurol [Internet]. 2020;16(2):97–107. Available from: http://www.ncbi.nlm.nih.gov/pubmed/31980808
6. Vollstedt E, Kasten M, Klein C, Aasly J, Adler C, Ahmad-Annuar A, et al. Using global team science to identify genetic Parkinson's disease worldwide. Annals of Neurology [Internet]. 2019 Aug 26;86(2):153–7. Available from: https://onlinelibrary.wiley.com/doi/abs/10.1002/ana.25514
7. Global Parkinson’s Genetics Program. GP2: The Global Parkinson’s Genetics Program. Mov Disord [Internet]. 2021;36(4):842–51. Available from: http://www.ncbi.nlm.nih.gov/pubmed/33513272

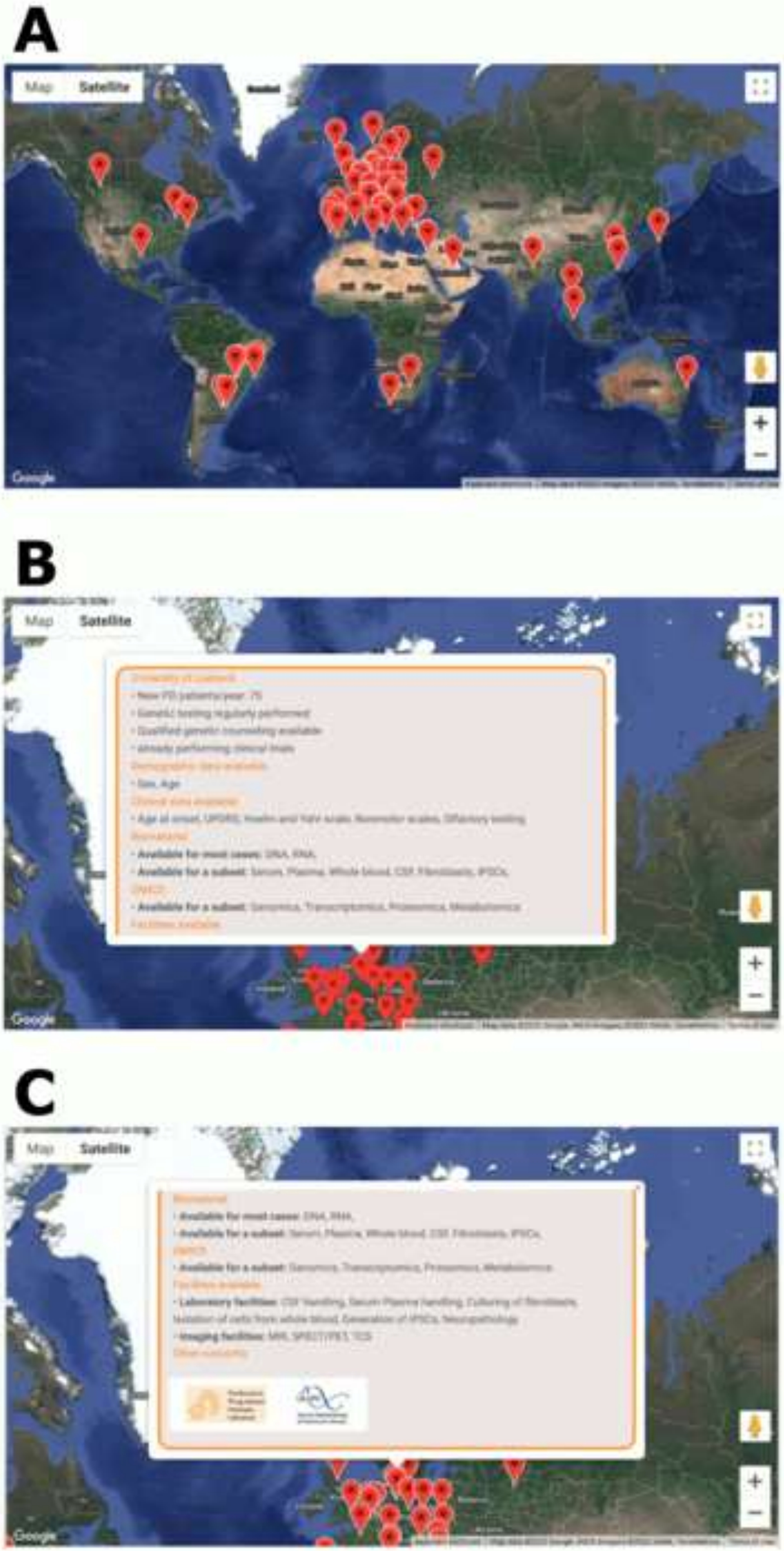
A
Map
Satellite
Google
B
Map
Satellite
Google
C
Map
Satellite
Google

Figure 2

Click here to access/download;Figure;Fig3.gif

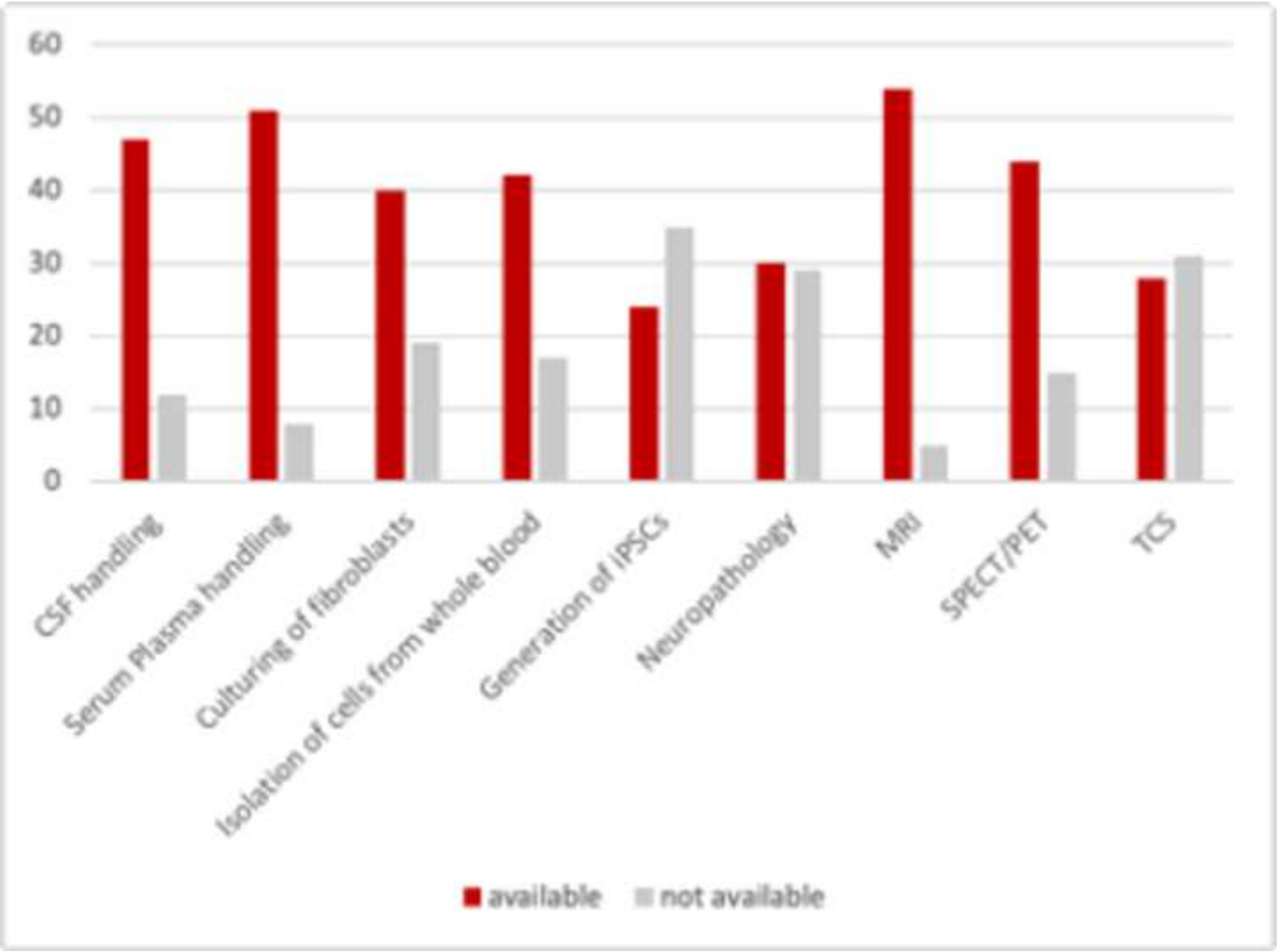

Figure 3

Click here to access/download;Figure;Fig2.gif

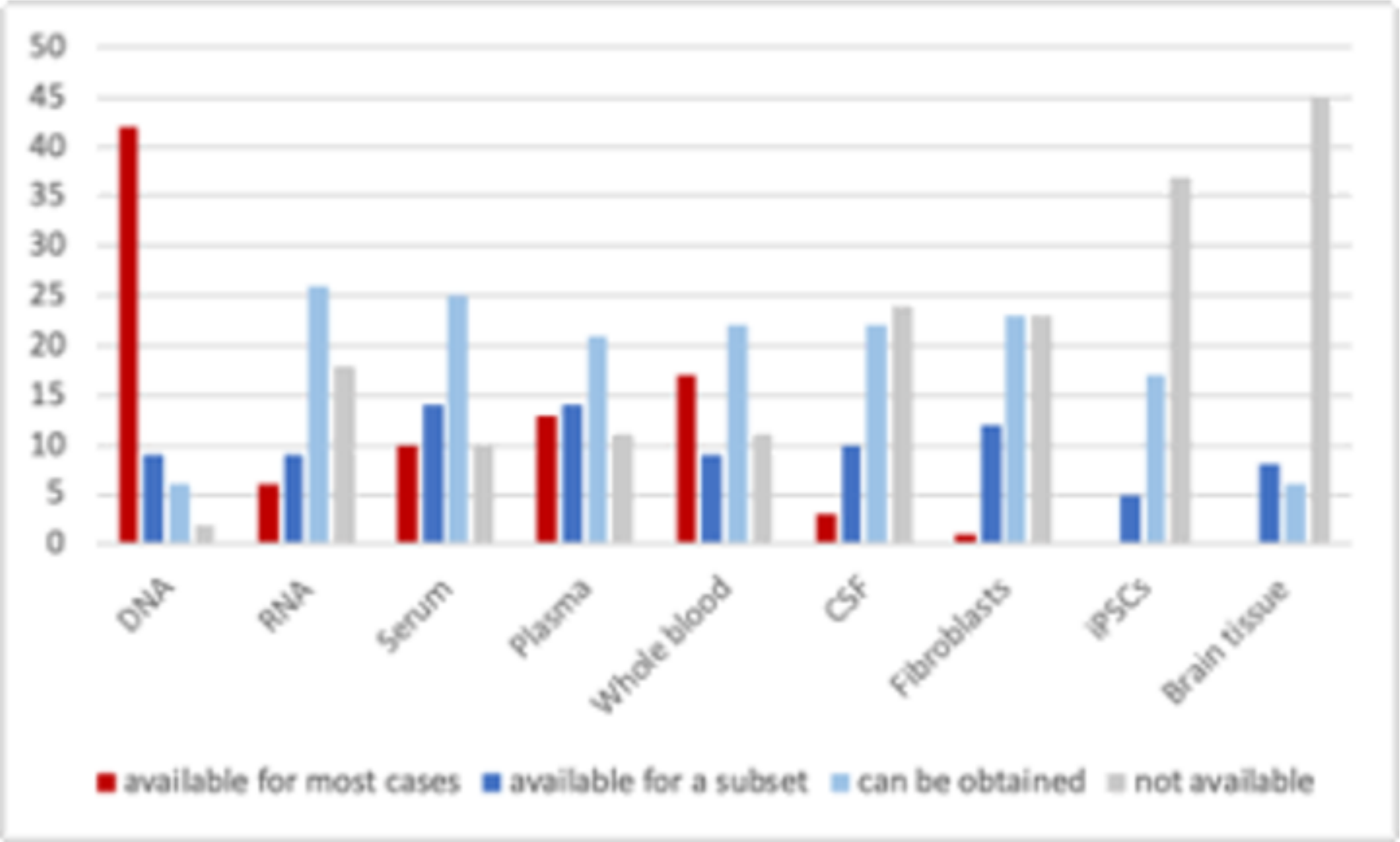

Supplement: Attachment — Submitted filename: PONE-D-22-35560_reviewer-2.pdf [file pone.0292180.s001.pdf]
