# Supplementary material for: Establishing an online resource to facilitate global collaboration and inclusion of underrepresented populations: Experience from the MJFF Global Genetic Parkinson’s Disease Project
Source: PLoS One. 2023 Oct 3;18(10):e0292180. doi: 10.1371/journal.pone.0292180 (PMC10547150; doi:10.1371/journal.pone.0292180)
Supplement: Attachment — Submitted filename: Rebuttal_PONE_Vollstedt_2023_09_01_CK.pdf [file pone.0292180.s003.pdf]

Dear Dr. Chenette,

We would like to thank you, Dr. El-Jaafary, and the anonymous Reviewer #3 again for the second review of our manuscript. We have addressed the reviewer's comment below and amended the manuscript accordingly.

Reviewer #3: *The authors have addressed the comments of the reviewers in detail and done so satisfactorily. The overall objective of the project described is to increase representation of under-represented populations. This flags one of the apparent shortcomings of the project which is that although a very significant population of URPs is in sub-Saharan black African populations across east, west and central Africa, the sites included in the efforts starkly exclude any sites from these African regions. Northern Africa (Tunisia) and Southern Africa are not typically regarded as underrepresented in terms of the ancestry of the population and their inclusion in genetic studies and one would presume that it would be important to have included other African regions. Indeed, excluding a significant underrepresented population in a project that seeks to enhance inclusion of underrepresented populations does seem counter-intuitive. The authors may wish to either clearly describe this as a limitation or, in implementing the initiative, address this drawback.*

Thank you very much for alluding to this misunderstanding. The reason for missing these centers is actually not active exclusion but lies in our initial approach to identifying suitable centers. For the initial step of this project, we contacted the corresponding authors of publications on genetic Parkinson's disease that we had systematically identified for the MDSGene project (www.mdsgene.org). MDSGene is a database including individual-level data on subjects with genetic causes for movement disorders that have been extracted from publications available in English. Unfortunately, with this approach, we likely missed centers that could have been included, as they may have published in languages other than English.

We strongly agree that the lack of centers with underrepresented populations is currently a limitation of this project. In order to further expand the network and to actively include underrepresented populations, this project is currently being integrated into the Global Parkinson's Genetic Program (GP2). GP2 specifically focuses on the inclusion of previously underrepresented populations and will consequently make more clinical and research centers visible and global study participants available to the clinical and basic PD research community. We have highlighted this limitation and outlook in the discussion section of our manuscript accordingly.

Thank you very much for your consideration.

C. Klein

Christine Klein, MD, FEAN
